# Characterization of Epizootic Hemorrhagic Disease Virus Serotype 8 in Naturally Infected Barbary Deer (*Cervus elaphus barbarus*) and *Culicoides* (Diptera: Ceratopogonidae) in Tunisia

**DOI:** 10.3390/v15071567

**Published:** 2023-07-18

**Authors:** Sarah Thabet, Soufien Sghaier, Thameur Ben Hassine, Darine Slama, Raja Ben Osmane, Ridha Ben Omrane, Wiem Mouelhi, Massimo Spedicato, Alessandra Leone, Liana Teodori, Valentina Curini, Moncef Othmani, Shadia Berjaoui, Paola Ripà, Makram Orabi, Bassem Belhaj Mohamed, Ayda Sayadi, Sonia Ben Slama, Maurilia Marcacci, Giovanni Savini, Alessio Lorusso, Salah Hammami

**Affiliations:** 1 Service de Microbiologie, Immunologie et Pathologie Générale, École Nationale de Médecine Vétérinaire de Sidi Thabet, IRESA, Université de la Manouba, Tunis 2020, Tunisia; sarahthabetbenabdeljelil@gmail.com (S.T.); saleehhammami@yahoo.fr (S.H.); 2 Institut de la Recherche Vétérinaire de Tunisie, Tunis 1006, Tunisia; sghaiersoufien@yahoo.fr; 3 Direction Générale des Services Vétérinaires, Commissariat Régional au Développement Agricole de Nbeul, Nabeul 8000, Tunisia; benhassinethameur@yahoo.fr; 4 Laboratory of Medical and Molecular Parasitology-Mycology LP3M (Code LR12ES08), Department of Clinical Biology B, Faculty of Pharmacy, University of Monastir, Monastir 5000, Tunisia; darineslama132@yahoo.fr; 5 Laboratoire National de Contrôle des Médicaments, Tunis 1006, Tunisia; raja.benosman@yahoo.com (R.B.O.); s.berjaoui@izs.it (S.B.); p.ripa@izs.it (P.R.); sayediaida7@gmail.com (A.S.); benslamasonia5@gmail.com (S.B.S.); 6 Direction Générale des Services Vétérinaires, Commissariat Régional au Développement Agricole de Ariana, Tunis 2010, Tunisia; benomraneridha@yahoo.fr; 7 Direction Générale des Services Vétérinaires, Commissariat Régional au Développement Agricole de Jendouba, Jendouba 8100, Tunisia; w.ie@hotmail.fr; 8 Istituto Zooprofilattico Sperimentale dell’Abruzzo e Molise, 64100 Teramo, Italy; m.spedicato@izs.it (M.S.); a.leone@izs.it (A.L.); l.teodori@izs.it (L.T.); v.curini@izs.it (V.C.); m.marcacci@izs.it (M.M.); g.savini@izs.it (G.S.); 9 Direction Générale des Services Vétérinaires, Commissariat Régional au Développement Agricole de Tozeur, Tozeur 2200, Tunisia; othmani.medvet@yahoo.fr (M.O.); ourabimakrem@yahoo.fr (M.O.); 10 Centre National de Veille Zoosanitaire (CNVZ), Kébili 4200, Tunisia; bassemvet@gmail.com

**Keywords:** EHDV-8, whole genome sequencing, deer, *Culicoides*, vectors, Tunisia

## Abstract

Epizootic hemorrhagic disease (EHD) is a *Culicoides*-borne disease of domestic and wild ruminants caused by EHD virus (EHDV). This virus circulates in multiple serotypes. In late September 2021, a novel strain belonging to EHDV-8 was reported in cattle farms in Central–Western Tunisia, and in the fall of 2022, the same virus was also detected in Italy and Spain. In the present study, we described EHDV-8 occurrence in deer and, a preliminary identification of the potential *Culicoides* species responsible for virus transmission in selected areas of Tunisia. EHDV-8 was identified in deer carcasses found in 2021 and 2022 in the national reserve of El Feidja, Jendouba, Northwestern Tunisia, and isolated on cell culture. Instead, insect vectors were collected in October 2021 only in the areas surrounding the city of Tozeur (Southern Tunisia) where EHDV-8 cases in cattle were confirmed. Morphological identification showed that 95% of them belonged to the *Culicoides kingi* and *Culicoides oxystoma* species and both species tested positive for EHDV-8 RNA. *C. imicola* was not detected in this collection and EHDV-8 RNA was not evidenced in vector pools collected in 2020, prior to official EHDV-8 emergence. EHDV whole genome sequences were also obtained directly from infected biological samples of deer and positive vectors. EHDV-8 sequences obtained from deer and vectors share a nucleotide identity ranging from 99.42 to 100% and amino acid identity from 99.18 to 100% across all genome segments with the EHDV-8/17 TUN2021 reference sequence.

## 1. Introduction

*Epizootic hemorrhagic disease virus* (EHDV) is an arthropod-transmitted virus of domestic and wild ruminants belonging to genus *Orbivirus* of the family *Sedoreoviridae* that primarily affects white-tailed deer (*Odocoileus virginianus*) in North America and cattle (*Bos taurus*) [1,2,3,4]. This virus causes a hemorrhagic disease (EHD) that may result in high mortality rates, particularly in white-tailed deer.

The virus is transmitted by biting midges of the genus *Culicoides* (Diptera: Ceratopogonidae) and is closely related to the bluetongue virus (BTV) (ICTV, https://ictv.global/report/chapter/sedoreoviridae, accessed on 4 March 2023). EHDV was first identified in white-tailed deer in the United States in the middle of the 20th century. Since then, the disease has been detected in several regions of the world [5]. EHDV infection in deer is associated with high fever, lethargy, oedema, ulcerations of the dental pad and oral mucosa, hemorrhaging of the heart, lungs, major blood vessels, and other tissues. EHDV genome comprises 10 linear segments of double-strand RNA, coding seven structural (VP1–VP7) and four non-structural (NS1–NS4) proteins [6]. The outer-capsid protein VP2 is the primary determinant of serotype specificity. Seven distinct but provisional serotypes are officially recognized (1, 2, 4–8) [7,8,9,10]. Indeed, the relationship between the individual serotypes has not been fully determined and serotype classification is challenging.

In the past two decades, there has been a gradual increase in the number of EHD outbreaks worldwide in cattle characterized by BTV-like clinical signs [11]. In Tunisia, the first EHD outbreak was described in 2006, caused by EHDV-6, which was responsible for significant mortality and morbidity as well as large economic losses for different cattle farms. EHDV-6 strains from Tunisia were closely related to EHDV-6 strains circulating in Morocco and Algeria [12].

In late September 2021, a novel strain belonging to EHDV-8 was reported in cattle farms in Central–Western Tunisia and then spread rapidly across the Northern and Eastern regions during October and November, with more than 200 confirmed outbreaks [11]. The same strain was then identified in the fall of 2022 in symptomatic cattle in Italy (Sardinia and Sicily islands) and Spain (Andalusia), posing a serious hazard for the livestock industry in Europe as no vaccines are currently available [13].

As for vectors, only certain species of the genus *Culicoides* (*C*) were demonstrated, historically, to be competent vectors of EHDV. In Africa, the major vector is the *C. Schultzei* midges group including *C. oxystoma*, *C. schultzei*, *C. subschultzei*, *C. kingi*, *C. rhizophorensis*, *C. enderleini*, *C. nevilli*, and *C. neoschultzei* [14,15]. In North America, the virus has been mainly isolated from *Culicoides sonorensis* [16]. In Australia, multiple EHDV isolations from *C. brevitarsis* have been reported [17]. Understanding the *Culicoides* species involved in the transmission of the novel EHDV-8 strain is essential to enable the identification of high-risk transmission area. In our previous report [11], we focused only on EHDV-8 characterization from cattle, on the description of its novel genome constellation, and the antigenic relatedness with EHDV-6. However, description of the disease in deer and data upon *Culicoides* species found to be EHDV-8-positive were not provided.

## 2. Materials and Methods

### 2.1. Ethical Statement

This study did not involve any animal experimentation. Organs from deer and blood samples from cattle were collected by the Tunisian Veterinary Services within the context of outbreaks’ investigation, following standard procedures; thus, no ethical approval was required.

### 2.2. Deer Necropsy and Sampling

**Figure 1:**
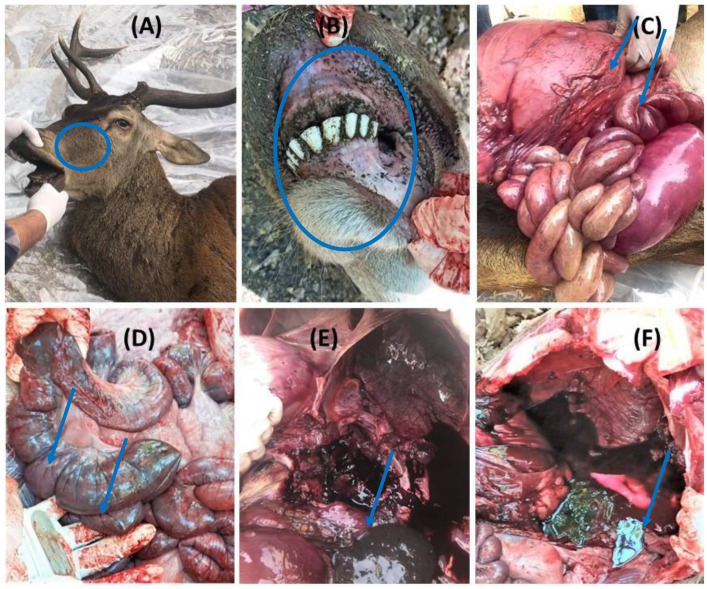
Lesions in two EHDV-8-positive Barbary deer; (**A**): congestion of the ocular mucosa; (B): cyanosis of the mouth; (**C**): generalized congestion of the digestive mucosa and liver; (**D**): hemorrhagic enteritis; (**E**): pulmonary hemorrhage; (**F**): hemorrhage in the abdominal cavity.

A total of seven Barbary deer, two in 2021 and five in 2022, were found dead in the national reserve of El Feidja Jendouba, Northwestern Tunisia, in the municipality of Ghardimaou. A total of three deer (one in 2021 and two in 2022) were necropsied. Biological samples were collected and shipped to the National Veterinary Research Institute (IRVT) the same day in a refrigerated cooler and conserved at −80 °C for diagnostic purposes. The remaining carcass from 2021 was necropsied but samples were not tested for further investigation; the samples of carcasses from 2022 were not processed due to the advanced state of putrefaction. Overall, the three deer carcasses showed similar *post mortem* lesions including congestion of the ocular mucosa, generalized congestion of the digestive mucosa and hemorrhagic lesions in the lungs (Figure 1A–C,E for deer 1 and Figure 1D,F for deer 2). Tissue samples from hemorrhagic organs including spleen, kidney, lung, and heart (twelve in total) were tested for the presence of BTV and EHDV RNA using molecular methods according to the epidemiological data collected upon *Orbivirus* circulation in the country [11].

### 2.3. RNA Purification and Molecular Tests

Total RNA purified from tissue samples and from *Culicoides* pools was extracted using the Pure Link Viral RNA/DNA miniKit (Invitrogen, Carlsbad, CA, USA). The RNA was eluted with 50 µL of ultrapure water and used as a template for the detection of BTV and EHDV RNA using real-time RT-PCR (RT-qPCR) [18] and VetMAX™ EHDV Kit Thermo Scientific™, Waltham, MA, USA), respectively. EHDV-positive samples underwent further testing by means of genotype-specific assays targeting the Seg-2 of all EHDV serotypes including a home-made specific RT-qPCR test for Seg-2 of EHDV-8 TUN2021 (RT-qPCR_seg-2 EHDV-8_) firstly identified in cattle in Tunisia in 2021 [11]. As for RT-qPCR_seg-2 EHDV-8_, primer nucleotide sequences were EHDV_Ser8varNEW_fwd AGAGATGAAGATCGCGAGGA and EHDV_Ser8varNEW_rev GAATCACACGCGCTCACTAA; the probe nucleotide sequence was EHDV_Ser8varNEW_Probe FAMACGGATGAGATACGGAACATACGGGG-TAMRA. A master mix using TaqMan Fast Virus 1-Step (Thermo Fisher Scientific) with a final concentration of 400 nmol of primers and 200 nmol of probe was prepared. Then, RNA denaturation at 95 °C for 3 min was performed, and 5 μL of RNA was added to 20 μL of mix. The amplification was set as follows: 45 °C for 10 min, 95 °C for 10 min, then 40 cycles of 95 °C for 15 s, and finally 60 °C for 1 min as previously described [13].

### 2.4. Virus Isolation

Virus isolation was performed starting from a total of six homogenized samples (mainly lungs, spleen, and heart) of deer that showed the lowest cycle threshold (CT) values. Virus isolation was performed by passing the tissue homogenates once onto KC (*Culicoides sonorensis* cell line) [19] confluent monolayers for ten days at 28 °C followed by two blind passages of Vero (African green monkey) cells at 37 °C, 5% CO_2_. Confirmation of EHDV-8 was obtained using RT-qPCR_seg-2 EHDV-8_. Furthermore, isolation directly on BSR, clone of baby hamster kidney-21 cells, was also attempted [20] using all the samples of deer.

### 2.5. Culicoides Collections

*Culicoides* midges were collected in October 2021 in a cattle farm in Tozeur (Southern Tunisia) where cases of EHDV-8 in cattle were confirmed. Midges were collected by means of an UV-Light/suction trap (OVI) manufactured by the Onderstepoort Veterinary Institute (South Africa) [21]. The trap was hung near the cattle at head height, sheltered from the wind and any artificial light source, and near a water pond. It was set in motion at dusk and stopped at dawn due to the twilight and nocturnal activity of the majority of *Culicoides* species. Midges collected in previous BTV surveillance activities from 2020, prior to official EHDV-8 detection, were used as negative controls. These midges originated from the municipalities of Jelma (Central Western Tunisia) and Hajeb (Central Tunisia). All captured specimens were preserved in 70° ethanol away from light.

### 2.6. Culicoides Discrimination by Morphological Analysis

The captured insects were sorted using a stereomicroscope. *Culicoides* midges were morphologically identified according to the wing patterns. The males were separated from the hematophagous females based on the shape of the genitalia observed in the last segments of the abdomen, which constitute an important taxonomic element [22].

*C. kingi* and *C. oxystoma* were differentiated from each other based on specific morphological features such as the pigmentation pattern of the wings, the length of the antennal segments, the shape of the male genitalia, and the posterior abdominal segment as previously described [23]. The parous and gravid females of each species were divided into pools of 15 individuals each, 11 pools of *C. kingi* and 13 pools of *C. oxystoma*. All pools were composed of parous and gravid females as follows: 10 pools of *C. kingi* and 9 pools of *C. oxystoma* from Tozeur, 1 pool of *C. kingi* and 1 pool of *C. oxystoma* from Jelma, and 3 pools of *C. oxystoma* from Hajeb.

### 2.7. Illumina Genome Sequencing

To characterize the genome constellation of EHDV-8 in deer and in *Culicoides* pools, five samples were selected for Illumina sequencing, three from deer and two from *Culicoides* (Table 1). Total RNA was treated and purified, as previously described, and then used for the assessment of SISPA protocol [24]. The PCR products were purified using ExpinTM PCR SV (GeneAll Biotechnology CO., Seoul, Korea) and then quantified using Fluorstar Omega (BMG LABTECH, Weston Parkway, Cary, NC, USA). Library preparation was performed using Illumina^®^ DNA Prep, (M) Tagmentation (96 samples, IPB) (Illumina Inc., San Diego, CA, USA) according to the manufacturer’s protocol. Sequencing was carried out on the NextSeq500 platform (Illumina Inc., San Diego, CA, USA) using the NextSeq 500/550 Mid Output Reagent Cartridge v2 (300 cycle) (Illumina Inc., San Diego, CA, USA) and standard 150 bp paired-end reads. After quality check and the trimming of raw reads data using FastQC v0.11.5 and Trimmomatic v0.36, respectively, host depletion was performed using Bowtie2 [25]. Obtained reads for each sample were mapped against the reference EHDV-8/17 TUN2021 sequence (acc. nos. OP381190-99). Consensus sequences were shared with the Genbank database and analyzed along with eight additional whole genome sequences of EHDV-8 obtained from infected blood collected from cattle in Tunisia in 2021 and 2022.

## 3. Results

### 3.1. EHDV-8 Was Identified and Isolated from Deer

All twelve samples from deer were positive for EHDV RNA, with CT values ranging from 20 to 30. They were also BTV negative. Samples were also demonstrated to be EHDV-8 TUN2021-positive and negative for the extant EHDV serotypes. A viral isolate was obtained from a total of four deer samples, three from spleens and one from the lungs. The isolation from deer samples was successful by combining KC cells and Vero cells, whereas isolation was not achieved when tissue homogenates were inoculated only in BSR cells.

### 3.2. EHDV-8 RNA Was Evidenced in Culicoides Kingi and Culicoides Oxystoma Pool Samples

The morphological identification of collected midges in Tozeur showed that 95% of them belonged to the *Culicoides kingi* and *Culicoides oxystoma* species. *Culicoides imicola*, the main vector of BTV in Tunisia, was not detected in the collection from this area, confirming previous and analogous findings (Soufien Sghaier, personal observation). *Culicoides imicola* was only detected in the collections performed in 2020 in Jelma and Hajeb. Pan RT-qPCR for EHDV RNA detection was performed on the pools of adult females belonging to *Culicoides kingi* and *Culicoides oxystoma* from Tozeur. They all tested positive for EHDV, with CT ranging from 16 to 35 (Table 2). Lastly, these samples were further typed as EHDV-8 TUN2021. In contrast, the pools from 2020 were all negative for EHDV RNA. All pools of *Culicoides kingi* and *Culicoides oxystoma* from 2020 and 2021 were also negative for BTV RNA.

### 3.3. Genome Analysis

All EHDV-8 sequences obtained from deer and vectors were shared within the Genbank database and the corresponding accession numbers are available in Table 1. Complete or nearly complete EHDV-8 consensus sequences were obtained from deer samples (sample ID 1-3, Table 1). As far as vectors are concerned, EHDV-8 sequences obtained from the *C. kingi* pool were partial (sample ID 4, Table 1) but identical to homologous and complete segments obtained from the pool of *C. oxystoma* (sample ID 5, Table 1). Overall, all sequences from deer and vectors share a nucleotide (nt) identity ranging from 99.42 to 100% and amino acid (aa) identity from 99.18 to 100% across all genome segmentswith EHDV-8/17 TUN2021 reference sequence. To identify whether any molecular signatures suggestive of host-specific adaption were present, all samples underwent genome analysis. Multi-alignment of nt and aa sequences were performed using Geneious Prime (Dotmatics, Boston, MA, USA). The aa sequences of proteins encoded by all ten segments of EHDV-8 strains identified in six cattle in 2021, including EHDV-8/17 TUN2021 reference sequence [11], were nearly identical with respect to each other as only three missense mutations were observed in Seg-4 (c.309G>A, p.Ala101Thr, reference sequence EHDV-8/17 TUN2021), in Seg-6 (c.938G>T, p.Val304Leu, sample ID 10) and in Seg-9 (c.691A>G, p.Asp228Gly, sample ID 13). The EHDV-8 genome sequence obtained from the single deer sampled in 2021 (sample ID 1) has unique mutations with respect to homologous sequences obtained from cattle of the same year, including one mutation in Seg-2 (p.Gln411Gly), two in Seg-3 (p.Thr247Ile and p.Asn817His) and one in Seg-9 (p.Gly125Arg). Two missense mutations, one in Seg-2 (p.Thr126Ala) and one in Seg-4 (p.Asn402Ser), were observed in deer sequences from samples collected in 2022. Scrutiny sequences obtained from the pool of *C. oxystoma*, three missense mutations including c.25C>T, p.Thr5Ile in Seg-1; c.24G>T, p. Asp3Tyr in Seg-3; and c. 736T>C, p.Ile243Thr in Seg-9, were evidenced with respect to the reference EHDV-8/17 TUN2021 sequence. C.24G>T, p.Asp3Tyr in Seg-3 was also present in sample ID 3 (deer of 2022), sample ID 6 and 8 (cattle 2022).

## 4. Discussion

In this study, we described the identification and molecular characterization of EHDV-8 in *Culicoides* midges and deer in Tunisia. We recently documented a large outbreak of EHD caused by a novel EHDV-8 strain which caused more than 200 confirmed cases in cattle across the country in 2022 [11]. The same strain has been also identified in the islands of Sardinia and Sicily (Italy) and in Andalusia (Spain), likely following pathways of introduction to Europe as described for several strains of different BTV serotypes [13]. Therefore, the characterization of EHDV-8-induced disease in hosts other than cattle and, importantly, the identification of the involved vector species are of the outmost importance. Indeed, in this study, we focused on Barbary deer, a species regularly found along the Tunisian–Algerian border [26], and in vectors collected in some areas in Tunisia where EHDV-8 circulation has been demonstrated. On the one hand, gaining knowledge on deer infection could be important to understand the role of this species in maintaining the disease in a given area; on the other, understanding which *Culicoides* species is involved in EHDV-8 transmission is crucial to assess the risk for other countries. Preliminary vector screening performed in a single area of Tunisia identified pools of *C. kingi* and *C. oxystoma* as being positive for EHDV-8. Reasonably, more accurate surveillance activities, and competence studies, need to be performed in further areas of Tunisia to ascertain the role of additional *Culicoides* species, in particular *C. imicola*, the main competent vector for BTV in this country. Importantly, pools of *Culicoides*, including *C. imicola*, collected in 2020, tested negative for the presence of EHDV-8 RNA. This could indicate that EHDV-8 was not circulating in the field prior to its emergence in 2021.

Next-generation sequencing techniques have completely changed the field of virus discovery and characterization. Indeed, these open novel avenues for the obtainment of insights into host-specific molecular signatures of pathogens that are widespread between different animal species. In modern molecular virology, understanding host adaptation mechanisms has been an important task to be accomplished since the establishment of the field; however, it was partially hindered either by the need for virus isolation, a procedure that could introduce mutations, including large deletions in the viral genome, or by Sanger sequencing technology, which is laborious and time-consuming. In this study, although on a limited number of samples, we analyzed the whole genome sequences obtained from EHDV-8-positive samples collected in two different time periods from at least three different hosts, including cattle, deer, and *Culicoides*. Overall, although sequencing was performed starting from infected biological samples, it was difficult assess a proper identification of host-specific molecular signatures as the number of processed samples was extremely low. Based upon the sequence data, we believe that only an increase in the number of single nucleotide polymorphisms and missense mutations in EHDV-8 genome sequences obtained from samples of 2022 was observed, a phenomenon which can likely be related to the evolution of a novel virus in naïve populations. Additional sequencing by enrolling a larger number of vector species collected from different locations will certainly help to disentangle the role of vectors in EHDV-8 evolution.

Our study has some limitations. First, we identified EHDV-8 RNA only in two *Culicoides* species, which represent just a small portion of the *Culicoides* diversity present in Tunisia. Therefore, additional collections and screening of vectors are highly recommended. We were not able, moreover, to characterize the infection kinetics in deer as only dead animals were processed. Disentangling deer infection could be important to assess the role of this species in virus persistence and evolution. Apparently, in the area under investigation, only Barbary deer were present. Infection in these individuals was likely acute due to the presence of hemorrhagic lesions in their carcasses, coupled with EHDV-8 molecular detection in internal organs. However, additional tests for other pathogens (e.g., for clostridiosis) were not carried out and no information on the time of the necropsy following death was provided; therefore, we cannot claim with certainty that the observed lesions were related only to EHDV-8 infection. Additionally, one could reasonably argue that the observed congestion and many of the apparent hemorrhages could result from the positioning of the dead animal and bacterial overgrowth after death. In this perspective, therefore, more accurate surveillance, or experimental infection of deer, need to be implemented to better describe the pathological pictures following EHDV-8 infection in this species.

In conclusion, more efforts are also warranted to understand the role of additional ruminant species, including sheep and goats, in the epidemiology of this emerging *Orbivirus*.

## Figures and Tables

**Table 1:** EHDV-8 sequences obtained from NGS analysis of deer, cattle and *Culicoides* samples. Results of EHDV RNA molecular detection and isolation attempts are also indicated. Accession numbers, GenBank accession numbers; S2, segment 2. DOC, date of collection; VI, virus isolation on KC/Vero cells; na, not attempted; +, VI-positive.

	Sample ID	Region	DOC	Host	Source	Pan-EHDV CT Values	EHDV-8 CT Values	Accession Nos	Strain Name	VI
**1**	Deer (2021)	Ghardimaou	26 October 2021	Deer	Spleen	20	21	OP897550-59	[EHDV-8/Deer TUN2021]	+
**2**	Deer 2 (2022)	Ghardimaou	10 August 2022	Deer	Liver	20	20	OP897560-69	[EHDV-8/Deer2 TUN2022]	-
**3**	Deer 1 (2022)	Ghardimaou	10 August 2022	Deer	Lung	21	23	OP971147-56	[EHDV-8/Deer1 TUN2022]	+
**4**	*Culicoides* 1	Tozeur	22 October 2021	*C. kingi*	Midges	24	23	Partial, not deposited	-	na
**5**	*Culicoides* 2	Tozeur	22 October 2021	*C. oxystoma*	Midges	20	20	OP937331-40	[EHDV-8/Culicoides sp/2 TUN2021]	na
**6**	NR1555	Jendouba	3 August 2022	Cattle	Blood	19	18	OP971117-26	[EHDV-8/Cattle Jendouba TUN2022]	+
**7**	NR1716	Bizerte	1 September 2022	Cattle	Blood	20	19	OP971127-36	[EHDV-8/Cattle Bizerte TUN2022]	+
**8**	NR1775	Tunis	8 September 2022	Cattle	Blood	22	21	OP971137-46	[EHDV-8/Cattle Tunis TUN2022]	+
**9**	NR2200 BV KKEBT 01	Kef	7 October 2021	Cattle	Blood	20	20	OP897500-09	[EHDV-8/19 TUN2021]	+
**10**	NR2212/2 BV 9158	Sidi El Heni	10 October 2021	Cattle	Blood	21	20	OP897510-19	[EHDV-8/30 TUN2021]	+
**11**	BV2246 7/10/21	Jelma	7 October 2021	Cattle	Blood	20	19	OP897520-29	[EHDV-8/42 TUN2021]	+
**12**	BV2247 7/10/21	Siliana	7 October 2021	Cattle	Blood	19	18	OP897530-39	[EHDV-8/43 TUN2021]	+
**13**	NR2267 BV T6	Thala	12 October 2021	Cattle	Blood	20	19	OP897540-49	[EHDV-8/60 TUN2021]	+

**Table 2:** Results of RT-qPCR Pan EHDV of pooled parous *Culicoides kingi* and *Culicoides oxystoma*. CT, threshold cycle. -, negative.

Origin	Pool ID	*Culicoides* Species	RT-qPCR Pan EHDV CT Values
**Tozeur** 22 October 2021	P1	*Culicoides kingi*	24
	P2	*Culicoides kingi*	32
	P3	*Culicoides kingi*	34
	P4	*Culicoides kingi*	28
	P5	*Culicoides kingi*	35
	P6	*Culicoides kingi*	28
	P7	*Culicoides kingi*	33
	P8	*Culicoides kingi*	27
	P9	*Culicoides kingi*	25
	P10	*Culicoides kingi*	28
	P11	*Culicoides oxystoma*	26
	P12	*Culicoides oxystoma*	20
	P13	*Culicoides oxystoma*	29
	P14	*Culicoides oxystoma*	26
	P15	*Culicoides oxystoma*	22
	P16	*Culicoides oxystoma*	26
	P17	*Culicoides oxystoma*	16
	P18	*Culicoides oxystoma*	25
	P19	*Culicoides oxystoma*	29
**Jelma** 17 September 2020	P20	*Culicoides kingi*	-
	P21	*Culicoides oxystoma*	-
**Hajeb** 16 September 2020	P22	*Culicoides oxystoma*	-
	P23	*Culicoides oxystoma*	-
	P24	*Culicoides oxystoma*	-

## Data Availability

Sequence data are available via Genbank. The accession numbers for the sequences produced are listed in Table 1.
